# A cross-sectional study of Victorian mobile intensive care ambulance paramedics knowledge of the Valsalva manoeuvre

**DOI:** 10.1186/1471-227X-9-23

**Published:** 2009-12-14

**Authors:** Gavin Smith, Malcolm J Boyle

**Affiliations:** 1 Ambulance Victoria, PO Box 2000, Doncaster 3108, Victoria, Australia; 2 Monash University, Department of Community Emergency Health and Paramedic Practice, PO Box 527, Frankston 3199, Victoria, Australia

## Abstract

**Background:**

The Valsalva Manoeuvre (VM) is a primary measure for terminating haemodynamically stable supraventricular tachycardia (SVT) in the emergency care setting. The clinical use and termination success of the VM in the prehospital setting has not been investigated to date. The objective of this study was to determine Melbourne Mobile Intensive Care Ambulance (MICA) Paramedic knowledge of the VM, and to compare this understanding with an evidence-based model of VM performance.

**Methods:**

A cross-sectional study in the form of a face-to-face interview was used to determine Melbourne MICA Paramedic understanding of VM instruction between January and February, 2008. The results were then compared with an evidence-based model of VM performance to ascertain compliance with the three criteria of position, pressure and duration. Ethics approval was granted.

**Results:**

There were 28 participants (60.9%) who elected a form of supine posturing, some 23 participants (50%) selected the syringe method of pressure generation, with 16 participants (34.8%) selecting the "as long as you can" option for duration. On comparison, one out of 46 MICA Paramedics correctly identified the three evidence-based criteria.

**Conclusions:**

The formal education of Melbourne's MICA Paramedics would benefit from the introduction of an evidence based model of VM performance, which would impact positively on patient care and may improve reversion success in the prehospital setting. The results of this study also demonstrate that an opportunity exists to promote the evidence-based VM criteria across the primary emergency care field.

## Background

The management of haemodynamically stable supraventricular tachycardia (SVT) in the form of atrioventricular nodal re-entry tachycardia (AVNRT) or atrioventricular re-entry tachycardia (AVRT) by Melbourne Mobile Intensive Care Ambulance (MICA) Paramedics has traditionally involved the use of vagal manoeuvres as a primary intervention, followed by pharmacological interventions. Recent changes to Victorian Ambulance clinical practice guidelines, which effectively inhibit pharmacological interventions (unless greater than 30 minutes from hospital), have created reliance upon the Valsalva Manoeuvre (VM) as the sole management method for these patients in the prehospital setting. Historically, VM education within the MICA paramedic course has been somewhat informal, and ongoing education relies heavily on cultural practice and individual learning.

A comprehensive literature review revealed no standardised prehospital VM method in clinical practice use. [1] This review did however highlight a number of studies which supported technique, performance and a biomechanical basis of the VM for the treatment of SVT. However, these studies were confounded by a plethora of definitions that inhibited clarity of either defined practice or efficiency. The literature gave rise to the three elements of an evidence-based model of VM performance (Posture, Pressure and Duration). [2-7]

Six identified clinical studies compared clinical efficacy of VM against other vagal manoeuvres, and highlighted the safety of the VM for prehospital use, whilst also suggesting that early intervention improves clinical outcome. [8,3,4-12]

### Biomechanics of the VM

The VM is characterised by four distinct phases of action, precipitated by onset of strain due to the generation of an increased intrathoracic pressure. Traditionally this has been against a closed glottis, but evidence suggests that an open glottis assists in prevention of potential deleterious side effects. [11,4,13] The four Phases of effect are as follows [6,11,2,13]:

• Phase 1: Transient increase in aortic pressure with compensatory decrease in heart rate due to increased Intrathoracic pressure.

• Phase 2: End of transient period, with decreasing aortic pressure and increasing heart rate.

• Phase 3: Decreasing aortic pressure and compensatory rise in heart rate (end of strain phase).

• Phase 4: Increased venous return leads to increased aortic pressure and compensatory decrease in heart rate.

### An evidence-based VM model

The three elements below are derived from biomechanical studies defining the optimum impact on vagal tone at varying stages of the VM (primarily Phase two and Phase four). This information was promulgated in the article by Taylor and Wong. [2]

• Posture (supine)

• Pressure (40 mmHg)

• Duration (15 seconds)

The posture of those performing the VM can best be described by Wong and Taylor, whose study demonstrates an increase in efficiency when the patient is supine through elimination of increased basal sympathetic tone present in an upright subject. [8] Singer et al also support the use of the supine position due to reduced basal vascular tone, accompanied by study results that demonstrate greater influence on falling blood pressure during phase two and overshoot in phase four of the VM when the patient assumes the sitting or standing position. [4]

Individual components of the VM, such as pressure generation (to an optimum of 40 mmHg) are also identified independently by Waxman et al and Mehta et al. [13,10] Looga also defines a pressure of at least 40 mmHg to attain appropriate maximisation of vagal tone whilst preventing overt sympathetic responses following the manoeuvre. [11]

The duration of the VM is also quantified by Looga, who describes a duration of 15 seconds, which encompasses all of the strain phases of the VM without prolongation of any one phase, thus maximising efficiency of the VM as a whole. [11]

As the evidence-based VM model [11,10,4,2,13,8] described above has not been investigated to date in the prehospital setting, the objective of this study was to determine Melbourne MICA Paramedic knowledge of the VM, and to compare this with an evidence-based model of practice.

## Methods

### Study Design

A cross-sectional study (in the form of a face-to-face interview) was used to determine Melbourne MICA Paramedic understanding of the VM.

### Process

Written advertisements were placed in Melbourne metropolitan MICA ambulance stations to recruit MICA Paramedics for a face-to-face interview to identify MICA Paramedic management of SVT. Each participant was presented with a clinical scenario of a haemodynamically stable patient with SVT sitting on the edge of a bed in a residence. Participants were then asked to verbally detail their method of instruction of the VM to the patient. The clinical scenario and survey tool was modelled on that used in the Taylor and Wong study following consultation with the authors. [2] Participants were blinded to the research question and purpose of the study. The data was collected using a paper-based survey, and the results were subsequently analysed and a comparison made to the evidence-based VM model. The data was collected between mid-January and the end of February, 2008.

### Setting

The study was conducted in Melbourne, Australia. The Metropolitan region of Ambulance Victoria (AV-M) provides the Emergency Medical Service (EMS) for the greater Melbourne metropolitan area which covers approximately 7,694 square kilometres and a population of some 3.81 million people. [14]

### Population

Melbourne has both a single and dual level Emergency Medical Service (EMS) response. The first level of EMS response is provided by an Ambulance Paramedic with varying levels of Advanced Life Support (ALS) skills. The second level of EMS response is the Mobile Intensive Care Ambulance (MICA) Paramedic who has a broader range of ALS skills including intubation, rapid sequence intubation, and a wider range of pharmacological interventions available. In the Melbourne metropolitan service area there were 230 operational MICA Paramedics eligible for the study. Inclusion criteria for the survey were a MICA Paramedic qualification and holding an operational position within AV-M. Student MICA Paramedics, ambulance paramedics, and those MICA Paramedics within AV-M holding non-operational, office based, positions were excluded to enable identification of existent practice within the cohort.

### Ethics

Ethics approval for the study was granted by the Monash University Standing Committee on Ethics in Research involving Humans and approval for the study with Ambulance Victoria MICA Paramedics was granted by the Ambulance Victoria Research Governance Committee.

### Analysis

Data analysis was undertaken using SPSS (Statistical Package for the Social Sciences Version 17.0, SPSS Inc., Chicago, Illinois, U.S.A.). Descriptive statistics were used to summarise the demographic and VM data.

## Results

A total of 46 MICA Paramedics volunteered, with a 100% participation rate to study completion, representing a 20% sample of the total Melbourne metropolitan operational MICA Paramedic workforce.

The MICA paramedic responses to the question of posture are contained within Table 1, with the largest proportion of responses (34.8%) selecting the "supine with feet elevated" option, whilst a lesser percentage (26.1%) elected "supine" posturing.

**Table 1:** Position for the Valsalva Manoeuvre

Position	n	%
Supine	12	26.1
Supine with feet elevated	16	34.8
Sitting	14	30.4
Semi-recumbent	4	8.7
Total	46	100.0

The majority of participants (34.8%) elected the "as long as you can" option for duration of strain (intra-thoracic pressure generation), The data survey form was modelled on the Taylor and Wong survey tool, resulting in the "11-15 second" option representing an approximation of the 15 seconds identified within the evidence-based VM model. Only 8 of the 46 participants (17.4%) reported this duration, as described in Table 2.

**Table 2:** Duration of the Valsalva Manoeuvre

Duration	n	%
<5 seconds	1	2.2
5-10 seconds	11	23.9
11-15 seconds	8	17.4
>15 seconds	7	15.2
As long as you can.	16	34.8
Other	3	6.5
Total	46	100.0

The results listed in Table 3 demonstrate that no paramedic participant elected to utilise a sphygmomanometer to record pressure generation, yet 23 of the 46 participants (50%) reported use of the syringe method to generate the required intrathoracic pressure.

**Table 3:** Method of Valsalva Manoeuvre

Method	n	%
Blow into a syringe	23	50.0
Strain like defecation	13	28.3
Blow against a closed glottis	3	6.5
Abdominal pressure	7	15.2
Total	46	100.0

Overall, MICA Paramedics were largely (65.2%) unable to provide a single element of the evidence-based model of VM performance, with only one of 46 participants (2.2%) stating the three necessary criteria for optimum performance, as demonstrated in Table 4.

**Table 4:** Comparison of MICA data with an evidence-based model of the VM

Element of evidence-based model	MICA data
No element	30 (65.2%)
One element	4 (8.7%)
Two elements	11 (23.9%)
Three elements	1 (2.2%)
Total	46 (100%)

## Discussion

The use of an evidence-based model of VM performance is an efficient, safe and inexpensive manner of attempting termination of SVT in the prehospital and emergency medicine setting. As there have been no previous efforts to determine an appropriate method of VM instruction in the prehospital setting, this model enables an evidence-based approach to maximising vagal tone (and hence the effect of the VM) when applied to patients with haemodynamically stable SVT. It also enables a uniform approach to the management of SVT in the prehospital setting which is likely to produce improved patient care outcomes.

The study of position as a component of VM demonstrated that the MICA Paramedic cohort was divided between the supine and sitting position. Although a majority of participants in this study chose to place the patient in a supine with feet elevated position, when coupled with the supine position this results in a large proportion of supine posturing (60.9%) overall. The 30.4% of respondents selecting seated posturing revealed an incomplete understanding of position in relation to vagal efficiency, and as a result would be more likely to encounter adverse side effects related to hypotension and syncope as a result. [8,4] The predisposition of MICA Paramedics to place patients supine with feet elevated appears, anecdotally, related to older concepts abounding within paramedic practice of the potential to increase venous return from the elevated legs.

The simplest quantifiable methods of attaining a pressure of 40 mmHg for VM performance in the prehospital and emergency medical setting have been identified as either the use of a sphygmomanometer [2,8,15], or the 10 ml syringe [16]. This aspect of the study elicited a high level of response from the MICA Paramedic group, with 50% electing to utilise the syringe. This result was somewhat expected, as this method has anecdotally been known in Victorian MICA Paramedic circles for some time as a means of pressure generation, though its efficiency has not been subject to testing until recently. [16] This cultural knowledge is also likely to have resulted in the MICA Paramedic cohort being more conscious of using a syringe rather than a sphygmomanometer to generate the required pressure as part of the VM generally.

The duration responses of the VM demonstrated by the MICA paramedic cohort in this study suggest an incomplete understanding of the impact of duration on vagal tone. This is evidenced by the variation evident in the results, with the largest percentile (34.8%) attributed to the "for as long as you can" option. The evidence-based recommendation of 15 seconds accounted for only 8 (17.4%) respondents. This incomplete understanding would likely translate into inefficiencies in practical application and reversion effect.

Comparison of the results of this study with an evidence-based model of VM performance (Table 4) demonstrated that the introduction of such a model would undoubtedly improve the standard of care provided to patients with haemodynamically stable SVT by MICA Paramedics through compliance with a means of maximising the effect of vagal manoeuvres in the prehospital setting. Of interest is that the results obtained demonstrate a trend toward a higher compliance to individual elements of an evidence-based model than a previously studied emergency physician cohort [2], suggesting the potential for this model to be incorporated into the wider primary care field for the management of SVT.

This study is potentially limited by the small sample size. The influence of cultural and individual learning to provide a higher than expected compliance with the evidence-based model is not quantifiable within this study, however further studies may be able to differentiate chance from acquired knowledge, and hence eliminate this potential limitation. The ability to generalise these results to the operational MICA Paramedic population in Victoria should be undertaken with caution as these results may not be a true representation of the total Victorian operational MICA Paramedic population.

## Conclusion

This study has highlighted a need to broaden and standardise the education of VM, through the promotion of an evidence-based model of practice, across the spectrum of primary emergency health care disciplines. At present, it would appear there is little scientific evidence utilised in the education of MICA Paramedics with regard to vagal manoeuvres and the reversion of SVT. This study has specifically identified the need for an evidence-based approach to the education of student MICA Paramedics, and a continuing education program for qualified MICA Paramedics, in the biomechanics and processes involved in terminating SVT in order to improve patient care.

## Competing interests

The authors declare that they have no competing interests.

## Authors' contributions

GS conceived the study and undertook the data collection. Both authors devised the study methodology. MB undertook the statistics and both authors compiled the manuscript. Both authors have read and approved the manuscript.

## Pre-publication history

The pre-publication history for this paper can be accessed here:

http://www.biomedcentral.com/1471-227X/9/23/prepub
